# Early versus Delayed Drug-Coated Balloons for Symptomatic Intracranial Atherosclerotic Stenosis

**DOI:** 10.1016/j.neurot.2025.e00689

**Published:** 2025-07-08

**Authors:** Xiaofei Sun, Yayue Liu, Lucynda Pham, Guangwen Li

**Affiliations:** a Department of Neurology, The Affiliated Hospital of Qingdao University, No.16 Jiangsu Road, Qingdao 266000, China; b Center for Molecular Medicine and Genetics, Wayne State University, Detroit, MI, 48201, USA

**Keywords:** DCB, ICAS, Stroke, TIA

## Abstract

In recent years, drug-coated balloons (DCB) have been increasingly used for intervention therapy of intracranial atherosclerotic stenosis (ICAS) with significant efficacy. However, the timing of DCB intervention therapy remains controversial, and there are few published studies that further investigated this. This study aimed to evaluate the clinical outcomes of early (≤21 days) versus delayed (21–42 days) DCB treatment in patients with ICAS. Symptomatic ICAS (sICAS) patients who underwent DCB angioplasty between August 2021 and March 2024 were included in the study. Based on the time from the last qualifying event (QE) to the procedure, patients were divided into an early group (≤21 days) and a delayed group (21–42 days). The efficacy and safety of DCB angioplasty, including perioperative complications and restenosis, were recorded and compared between the two groups. A total of 186 patients were enrolled, with 75 in the early group and 111 in the delayed group where all patients underwent DCB angioplasty successfully. The delayed group showed significantly lower postoperative residual stenosis (10 ​% vs. 20 ​%, P ​= ​0.041) and restenosis rates (10.81 ​% vs. 22.67 ​%, P ​= ​0.029) at the 12-month follow-up compared to the early group. The delayed group also had numerically lower perioperative complication rates (5.41 ​% vs. 9.33 ​%, P ​= ​0.303) and recurrence rates (7.21 ​% vs. 9.33 ​%, P ​= ​0.601), however these differences were not statistically significant. Our study concludes that in patients with sICAS, performing DCB angioplasty within 21 days may carry a higher degree of residual stenosis and an increased long-term risk of restenosis.

## Introduction

Intracranial atherosclerotic stenosis (ICAS) is a chronic vascular disease characterized by lipid deposition, inflammatory responses, and plaque formation in cerebral arteries. It is a major cause of ischemic stroke, particularly in Asian populations [1]. Current guidelines recommend standard medical therapy (including anti-platelet therapy, intensive lipid-lowering, and strict blood pressure/blood sugar control) for stroke prevention in ICAS patients. However, despite aggressive medical treatment, some patients remain at high risk of recurrent stroke, about 7.2 ​%–15.1 ​% per year [[2], [3], [4]]. In this case, intervention therapy plays an important role.

The Balloon Angioplasty for Symptomatic Intracranial Artery Stenosis (BASIS) trial reported that balloon angioplasty plus aggressive medical management may be an effective treatment for ICAS [5], and several studies have shown that drug-coated balloon (DCB) is superior to the conventional balloon [[6], [7], [8]]. The balloon is coated with anti-proliferative drugs (e.g., paclitaxel or sirolimus) and delivered to the lesion site via a catheter, followed by balloon dilation and drug release onto the blood vessel wall, thereby inhibiting endometrial hyperplasia and preventing restenosis [6,9]. However, the optimal timing for DCB intervention remains debated. A previous study by our team showed that the total rates of any ischemic stroke (IS), transient ischemic attack (TIA), hemorrhagic stroke, and death in the early stenting group (within 14 days) was higher than in the late stenting group in patients with ICAS, and the recurrence rate of ischemic stroke was also higher. These results suggest that stent placement in IS caused by ICAS within 14 days may confer a higher risk of long-term cerebral vascular events and lead to a higher risk of restenosis [10]. Some researchers argue that intervention within 21 days increases the risk of stroke and death, therefore delaying intervention may achieve better safety and efficacy [11]. Yu et al. found that the perioperative and post-operative stroke or death rates decreased with the time of stenting to the qualifying events. The perioperative stroke rate was significantly higher in patients treated within 21 days after the qualifying event, compared to those beyond 21 days. Similar relationships were obtained for both post-procedural and 1-year stroke or death rate [12]. In addition, Barnard and Alexander discovered that with a mean time to stenting treatment of 21 days or longer post-stroke, they demonstrated significantly lower periprocedural complication rates [13]. Nevertheless, there is limited literature and data relevant to the timing of DCB angioplasty. Therefore, this study aimed to compare the outcomes of early (≤21 days) and delayed (21–42 days) DCB intervention in symptomatic ICAS (sICAS) patients.

## Materials and Methods

### Study design

This study is registered at ClinicalTrials.gov (ChiCTR2100047223 and ChiCTR2300069547). This study included patients who underwent intracranial DCB intervention for ICAS-induced IS or TIA at the Affiliated Hospital of Qingdao University between August 2021 and March 2024. The study was approved by the hospital's ethics committee with written and oral informed consent obtained from all patients or guardians in accordance with the Declaration of Helsinki. Stenosis was confirmed using digital subtraction angiography (DSA), and the diagnosis and treatment of stenosis was confirmed by a central review committee of experienced neurologists, neurointerventionists, and radiologists. Early DCB was defined as intervention within 21 days of the last ischemic symptom, while delayed DCB was defined as intervention 21–42 days after the last ischemic symptom.

### Participants

The patients met the following inclusion criteria: Age 18–80 years; target vessels included intracranial carotid artery, middle cerebral artery, vertebral artery V4 segment, and basilar artery, with 70–99 ​% stenosis confirmed by DSA; target lesion length ≤15 ​mm, diameter ≥2 ​mm; presence of ischemic stroke symptoms caused by ICAS within 42 days before enrollment including TIA or IS.

The main exclusion criteria were as follows: Patients with large cerebral infarction, severe vascular tortuosity or calcification; non-atherosclerotic lesions, such as the presence of moyamoya disease, intracranial tumors, aneurysms or arteriovenous malformations, and emergency arterial embolism. An executive committee reviewed all clinical and imaging data to determine eligibility.

### Procedure

All patients received standard medical therapy, including aspirin (100 ​mg/day) and clopidogrel (75 ​mg/day) for 5 days before the procedure or a 300 ​mg clopidogrel loading dose preoperatively. Procedures were performed under general anesthesia. Conventional balloon pre-dilation (Tonbridge Medical, China) was performed before DCB delivery. The diameter of pre-dilation balloon was 50–80 ​% of the diameter of the normal blood vessels and the length completely covered both ends of the lesion. The pre-dilation balloon was inserted and inflated slowly to the nominal pressure and remained inflated for 30s followed by the balloon emptying slowly. The diameter of the DCB was equal to or larger than that of the pre-dilation balloon but did not exceed the reference vessel diameter, and its length exceeded the stenotic lesion by 2–3 ​mm. The DCB (Yinyi Biotech, China or Hemo, China) was advanced to the stenotic segment with a micro-guidewire and pressure increased gradually at 1 ​atm/s to the nominal pressure, and kept inflated for 60 ​s to deliver paclitaxel onto the vessel wall. After the DCB was removed, the lumen was evaluated by angiography to detect vascular dissection, perforation, and distal embolization. Technical success was defined as residual stenosis <50 ​%. Remedial stenting was performed if residual stenosis exceeded 50 ​% or dissection occurred. A complete neurological examination and head computerized tomography (CT) were conducted to rule out ischemic stroke or hemorrhage.

### Postprocedural management and follow-up

Dual antiplatelet therapy (aspirin 100 ​mg/day and clopidogrel 75 ​mg/day) was maintained for 3 months, followed by monotherapy with aspirin or clopidogrel. Risk factor management included blood pressure, glucose and lipid control, weight management, smoking cessation, and lifestyle modifications. All patients were followed up for 12 months and underwent DSA or Magnetic Resonance angiography (MRA), CT angiography (CTA) and Transcranial Doppler (TCD) 12 months after surgery. The described data were subsequently collected and recorded.

## Outcomes

Restenosis was defined as >50 ​% stenosis within or adjacent (≤5 ​mm) to the treated segment. Recurrent ischemic events included focal neurological symptoms in the corresponding vascular territory. Symptomatic restenosis was defined as recurrent stenosis with ischemic symptoms.

### Statistical analysis

Using the SPSS 25.0 software (IBM, Armonk, New York, USA) to analyze data, The Shapiro-Wilk test was used to analyze the data for normality. Normally distributed continuous variables are presented as mean ​± ​standard deviation (SD), while abnormally distributed continuous variables were expressed as median (IQR). Independent samples *t*-test and Mann–Whitney *U* test was used to identify differences in continuous variables respectively. Categorical variables were expressed as frequency and constituent ratio and compared using the χ^2^ test or the Fisher exact test. The exact *P*-value was calculated and P ​< ​0.05 was considered statistically significant.

## Results

### Baseline characteristics

We enrolled 186 patients with sICAS treated with DCB, divided into early (n ​= ​75) and delayed (n ​= ​111) groups. The patients’ baseline characteristics are shown in Table 1. Baseline characteristics (age, sex, BMI, hypertension, diabetes, hyperlipidemia, smoking history, and prior stroke) showed no significant differences. Preoperative stenosis severity, lesion length, MRS score, NIHSS score, and target vessel location were also comparable.

**Table 1:** Baseline characteristics of the patients.

Variables	Early DGB group (n ​= ​75)	Delayed DGB group (n ​= ​111)	P value
Age,years, mean (SD)	63.55 ​± ​9.42	60.85 ​± ​8.90	0.052
Gender, male,n (%)	50 (66.67 ​%)	74 (66.67 ​%)	1.000
BMI,mean (SD)	25.16 ​± ​2.68	25.89 ​± ​3.36	0.117
Hypertension, n (%)	61 (81.33 ​%)	91 (81.98 ​%)	0.911
Diabetes, n (%)	30 (40.00 ​%)	48 (43.24 ​%)	0.660
Hyperlipidemia, n (%)	28 (37.33 ​%)	40 (36.07 ​%)	0.611
Smoking history, n (%)	21 (28.00 ​%)	43 (38.74 ​%)	0.130
Stroke history, n (%)	30 (40.00 ​%)	43 (38.74 ​%)	0.863
Stenosis before procedure, %, median (IQR)	90.00 (80.00,90.00)	90.00 (80.00,90.00)	0.405
MRS score, median (IQR)	1 (0,1)	1 (0,1)	0.089
NIHSS score, median (IQR)	0 (0,1)	0 (0,11)	0.845
Lesion length, mm, mean (SD)	7.544 ​± ​3.1	7.344 ​± ​2.9	0.855
Qualifying artery, n (%)			
Internal carotid artery	16 (21.33 ​%)	25 (22.52 ​%)	0.848
Middle cerebral artery	25 (33.33 ​%)	42 (37.84 ​%)	0.530
Vertebral artery	17 (22.67 ​%)	21 (18.92 ​%)	0.534
Basilar artery	17 (22.67 ​%)	23 (20.72 ​%)	0.751

BMI ​= ​body mass index, MRS ​= ​modified Rankin Scale, NIHSS = National Institute of Health Stroke Scale.

### Procedural characteristics

During surgery, all balloons were successfully delivered to the lesion location and dilated. The delayed group had significantly lower residual stenosis (10 ​% vs. 20 ​%, P ​= ​0.041). Of the two groups, a total of 7 patients in the early group developed perioperative complications, including 3 cases of intracranial hemorrhage (ICH), 1 vascular dissection, 2 perforation and 1 case of thrombosis. Within the delayed group, 5 complications occurred, including 2 cases of ICH, 2 cases of perforation and 1 case of thrombosis. The incidence of perioperative complications in the delayed group was numerically lower than that in the early group, but the difference was not statistically significant (9.33 ​% vs. 4.50 ​%, p ​= ​0.203). A total of two patients underwent remedial stenting—one in the early group due to dissection and one in the delayed group due to residual stenosis >50 ​% after DCB angioplasty. No significant difference in remedial stenting rates was observed between the two groups (1.33 ​% vs. 0.9 ​%, p ​= ​1.000) (Table 2). **Clinical Follow-up**.

**Table 2:** Comparison of procedural features.

Variables	Early DGB group (n ​= ​75)	Delayed DGB group (n ​= ​111)	P value
Residual stenosis after procedure, %, median (IQR)	20.00 (10.00,30.00)	10 (10.00,20.00)	0.041
Perioperative complications, n (%)	7 (9.33 ​%)	5 (4.50 ​%)	0.203
ICH	3 (4.00 ​%)	2 (1.80 ​%)	0.394
Dissection	1 (1.33 ​%)	0 (0.00 ​%)	0.403
Perforation	2 (2.67 ​%)	2 (1.80 ​%)	1.000
Thrombosis Remedial stenting, n (%)	1 (1.33 ​%) 1 (1.33 ​%)	1 (0.90 ​%) 1 (0.90 ​%)	1.000 1.000

ICH = Intracranial hemorrhage.

Table 3 shows the long-term follow-up results of the early group and the delayed group. A total of 186 patients received DCB intervention therapy for 12 months where no death occurred. A total of 29 patients had restenosis degree ≥50 ​%, including 17 patients in the early group (22.67 ​%) and 12 patients in the delayed group (10.81 ​%), with statistically significant differences between the two groups (p ​= ​0.029). Patients with restenosis ≥70 ​% showed no difference (9.33 ​% vs. 9.01 ​%, P ​= ​0.940). In addition, a total of 15 patients had recurrent ischemic stroke events including 7 patients in the early group where 5 patients presented with IS and 2 with TIA. Compared to 8 patients in the late group, including 5 patients with IS and 3 patients with TIA. However, the difference was not statistically significant (9.33 ​% vs. 7.21 ​%, p ​= ​0.601). Finally, 12 patients had symptomatic restenosis, with 6 cases in each group (8.00 ​% vs. 5.41 ​%, p ​= ​0.549).

**Table 3:** Comparison of follow-up clinical outcomes.

Variables	Early DGB group (n ​= ​75)	Delayed DGB group (n ​= ​111)	P value
Degree of restenosis≥50 ​%, n (%)	17 (22.67 ​%)	12 (10.81 ​%)	0.029
Degree of restenosis≥70 ​%,n (%)<	7 (9.33 ​%)	10 (9.01 ​%)	0.940
Recurrence, n (%)	7 (9.33 ​%)	8 (7.21 ​%)	0.601
IS	5 (6.67 ​%)	5 (4.50 ​%)	0.527
TIA	2 (2.67 ​%)	3 (2.70 ​%)	1.000
Symptomatic restenosis≥50 ​%, n (%)	6 (8.00 ​%)	6 (5.41 ​%)	0.549

IS ​= ​ischemic stroke, TIA ​= ​transient ischemic attack.

## Discussion

To our knowledge, this is the first study comparing early (≤21 days) and delayed (21–42 days) DCB angioplasty for sICAS. Our key findings from this study include [1] early DCB increased long-term restenosis risk and [2] delayed DCB yielded lower residual stenosis.

In previous reports from the SAMMPRIS Trial, it was noted that performing intervention treatment within 7 days after IS carried higher risks of cerebral hemorrhage and hyper-perfusion syndrome. Conversely, delaying intervention until 30 days or later resulted in increased stroke recurrence rates, worsening stenosis (including progression into occlusion), thrombus formation, or collateral circulation deterioration [2] — findings consistent with results from the WASID Trial [14]. Additionally, Gao et al. observed in the CASSISS Trial that the optimal window for balancing risks and benefits was when intervention treatment was performed between 7 and 30 days after IS onset [4].

This study revealed a novel finding in which the timing of intervention significantly impacts post-procedural residual stenosis. Previous research has identified factors such as longer lesion length, severe calcification, lesion location, inadequate pre-dilation, and insufficient post-dilation as contributors to residual stenosis [15,16]. However, our results demonstrate that delaying intervention until at least 21 days after the qualifying event reduces the degree of residual stenosis. This observation warrants further validation through larger-scale studies.

Compared to some patients who had surgery within 21 days of qualifying events, patients in the delayed group may have had better preoperative preparation, such as dual antiplatelet therapy and risk factor control. At the same time, plaque gradually stabilizes over time, which may be related to plaque healing mechanisms [17]. Debris from unstable plaque fragmentation may be responsible for the higher degree of postoperative residual stenosis and long-term restenosis. In addition, after ischemic stroke, the repair and proliferation of vascular intima are active in the early stage [[18], [19], [20], [21]], and early intervention therapy may further stimulate this process. This may lead to excessive vascular remodeling, leading to a higher degree of postoperative residual stenosis and long-term restenosis.

Previous studies have suggested that the time interval from qualifying event (QE) to intervention may impact the outcomes. Performing intervention therapy more than 3 weeks after the QE may be safer than in less than 3 weeks after the QE [11]. Further supporting this, studies performing intervention more than 3 weeks after the QE have found lower rates of short-term death or stroke [4]. Patients who had carotid stenting delayed (>14 days) from the QE had a lower 30-day stroke and mortality than those who had the procedure within 14 days [18]. Jia B et al. found that the time from last symptom to surgery <18 days was a risk factor for perforator artery stroke [20]. Similarly, Rice CJ et al. reported that perioperative stroke rates were higher in patients with IS or TIA who underwent extracranial-intracranial bypass within 7 days [19]. The higher incidence of perioperative complications in early surgery may be related to the “snowplow” effect (plaque displacement to perforating artery) and reperfusion hemorrhage caused by plaque and intracranial microcirculation instability [11,21]. Moreover, the blood-brain barrier breaks down after ischemic damage to brain tissue [17,22]. Arteries and brain tissue do not have enough time to heal and as a result, there is an increased risk of overperfusion and cerebral hemorrhage [9,10]. In this study, the early group exhibited numerically higher rates of perioperative complications and long-term stroke recurrence compared to the delayed group, although the difference did not reach statistical significance. Nevertheless, in light of the literature discussed above, these findings suggest that early intervention may carry elevated risks and delaying intervention may provide better protection.

In patients with sICAS, performing DCB angioplasty within 21 days may carry a higher degree of residual stenosis and an increased long-term risk of restenosis.

## Limitations

First limitation of the study is this single-center study only recruited patients from China, and the selection of surgical equipment was based on patient conditions and surgeon experience, which may lead to selection bias. Second, although patients in the early group in this study had a higher probability of perioperative complications, recurrence and symptomatic restenosis than those in the delayed group, the results were not statistically significant, which may be due to the small sample size. Finally, although there was no difference between the two groups on the baseline data regarding the vessel where the lesion was located, the clinical outcomes of the corresponding vessels in the two groups were not analyzed separately, which warrants further subgroup analysis in the future.

## Ethics approval and consent to participate

This study involves human participants and was approved by the Medical Ethics Committee of the Affiliated Hospital of Qingdao University (ChiCTR2100047223 and ChiCTR2300069547). Participants gave informed consent to participate in the study before taking part.

## Consent for publication

Not applicable.

## Availability of data and materials

Data are available upon reasonable request. Data are available from the corresponding author upon reasonable request.

## Author contributions

XS drafted and revised the paper, conceived and designed the study, acquired and analyzed the data, applied software and statistics. YL acquired data. LP reviewed and edited the paper. GL undertook the study of patients and supervised the whole study, revised the paper and is responsible for the overall content as guarantor. All authors read and approved the final manuscript.

## Funding

This study was supported by the 10.13039/501100014761Natural Science Foundation of Qingdao (Grant no. 23-2-1-186-zyyd-jch).

## Declaration of competing interest

The authors declare that they have no competing interests.
